# Various techniques for resolving overlapping ultraviolet spectra of combination pharmaceutical dosage forms containing hydroxychloroquine and paracetamol

**DOI:** 10.1186/s13065-024-01187-2

**Published:** 2024-05-28

**Authors:** Samar S. Elbaramawi, Sobhy M. El-Adl, Alaa Nafea, Amr A. Mattar, Mahmoud M. Sebaiy

**Affiliations:** 1 Medicinal Chemistry Department, Faculty of Pharmacy, Zagazig University, Zagazig, 44519 Egypt; 2 Pharmaceutical Medicinal Chemistry Department, Faculty of Pharmacy, Egyptian Russian University, Badr, 11829 Cairo Egypt

**Keywords:** Hydroxy chloroquine, Paracetamol, Zero Crossing, Spectrum Subtraction, Dual Wave Length, Advanced Absorption Subtraction, Simultaneous Equation, Bivariate, Q-absorbance, Zero Crossing First Derivative, Ratio Difference and Ratio Derivative

## Abstract

Ten novel spectrophotometric approaches were developed for the initial examination of the Hydroxychloroquine and Paracetamol medications. These procedures are straightforward, specific, easy to use, and provide exact and accurate results. The determination was conducted through the utilization of several approaches, including zero order (dual wavelength, zero crossing, advanced absorption subtraction and spectrum subtraction), derivative (first derivative of zero crossing), ratio (ratio difference, ratio derivative) and mathematical (bivariate, simultaneous equation, and Q-absorbance) techniques. After undergoing validation in accordance with ICH criteria, it was established that each of these methods achieved acceptable levels of precision, repeatability, robustness, and accuracy. The advantages and disadvantages of each method are demonstrated, and the proposed and reported methodologies were statistically compared.

**Graphical Abstract:**

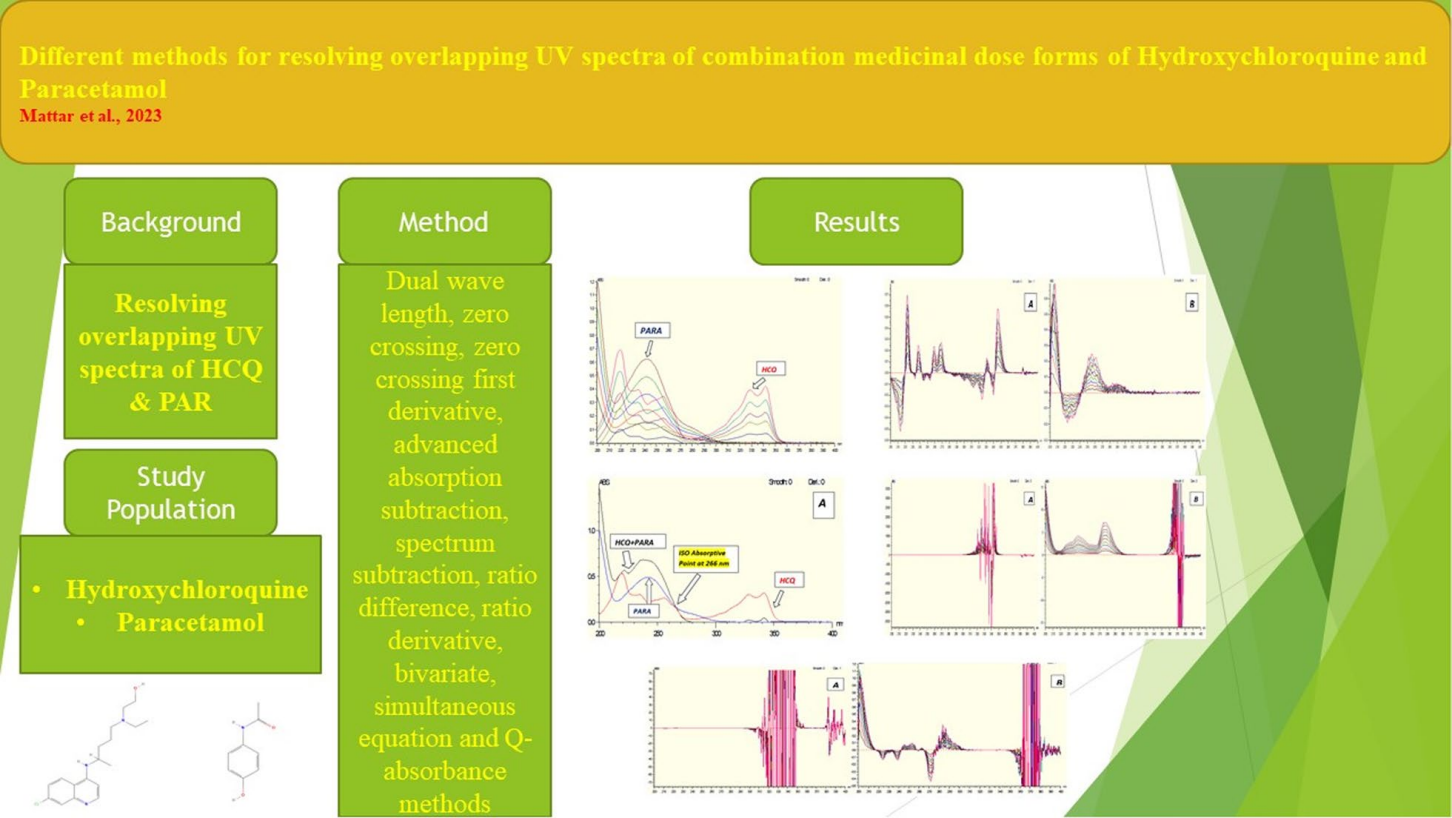

**Supplementary Information:**

The online version contains supplementary material available at 10.1186/s13065-024-01187-2.

## Introduction

Hydroxychloroquine and paracetamol are medications that have been used for a long time. The most common use of hydroxychloroquine is for the treatment and prophylaxis of malaria. However, this antimalarial drug is known to also have anti-inflammatory and antiviral effects and is used for several chronic diseases such as systemic lupus erythematosus with low adverse effects. The antiviral action of hydroxychloroquine has been a point of interest to different researchers due to its mechanism of action. Several in vitro studies have proven their effectiveness on severe acute respiratory syndrome virus. These investigations include the use of hydroxychloroquine and paracetamol, which is known for its pain-relieving (analgesic) and fever-reducing (antipyretic) properties [1, 2]. Some drugs were applied to nano-structured lipid carriers and niosomal systems [3]. The purpose of this article is to analysis of Hydroxy chloroquine and Paracetamol in a combined state, without prior separation.

**Fig. 1:**
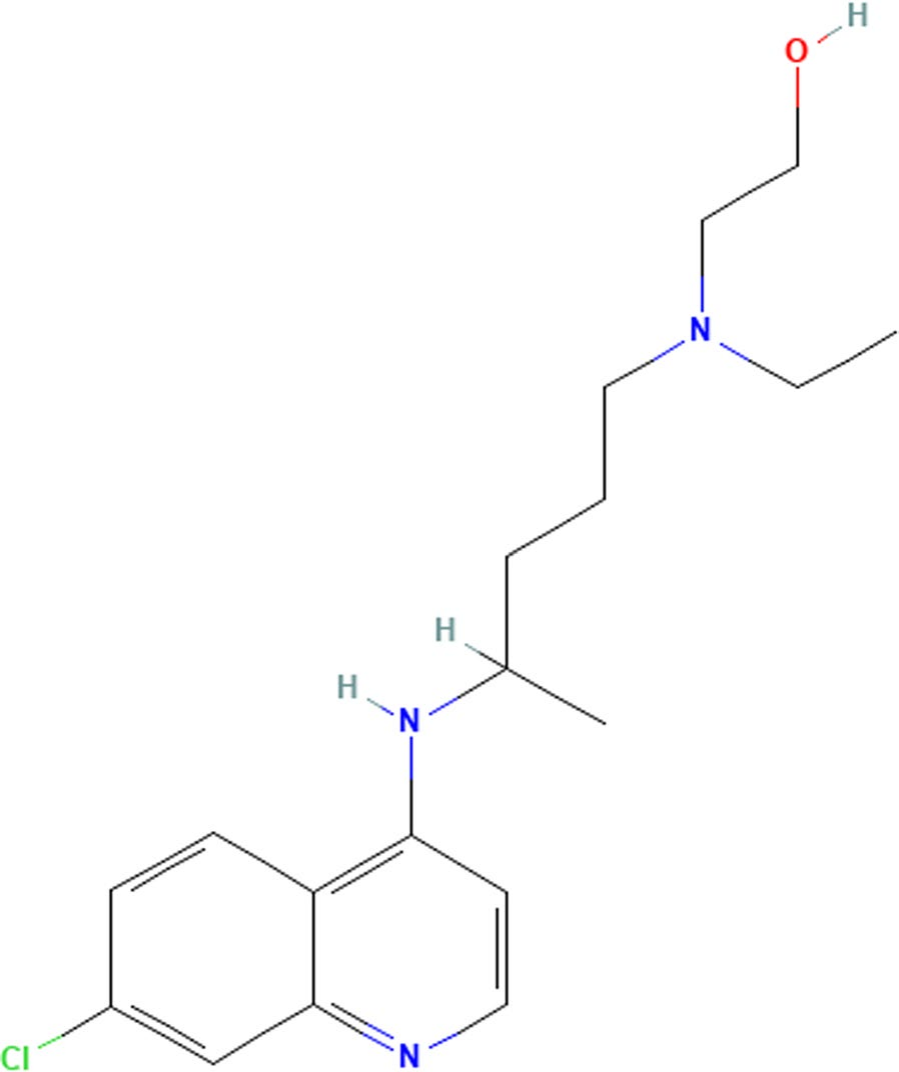
Chemical structures of Hydroxy Chloroquine (HCQ)

Paracetamol (PAR); N-(4-Hydroxyphenyl) acetamide (Fig. 1) is a medication that belongs to the class of non-steroidal anti-inflammatory drugs (NSAIDs). It works in both the central and peripheral nervous systems to treat non-inflammatory disorders [4]. In general, Acetaminophen is a widely used nonprescription analgesic and antipyretic medication for mild-to-moderate pain and fever has been stated to be the first-line analgesia according to the World Health Organization (WHO) pain ladder. It remains recommended as the first line of analgesia and antipyretic for patients [5–8].

**Fig. 2:**
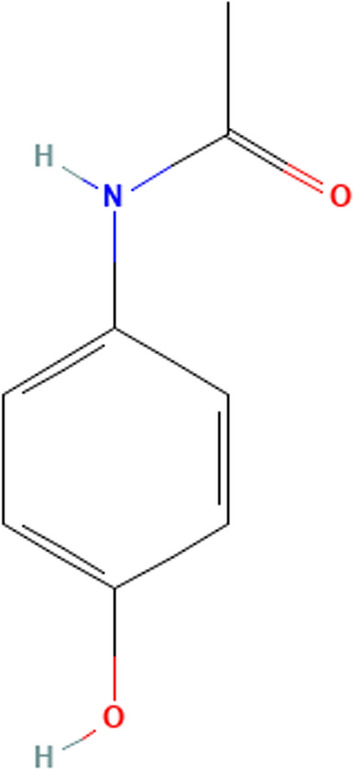
Chemical structures of Paracetamol (PAR)

Hydroxy chloroquine (HCQ); 2- [4- [(7-chloroquinolin-4-yl)amino]pentyl-ethylamino]ethanol (Fig. 2) Hydroxychloroquine is a derivative of chloroquine that has both antimalarial and anti-inflammatory activities and is now most often used as an anti-rheumatologic agent in systemic lupus erythematosus and rheumatoid arthritis [9, 10]**.**

Various techniques for assessing the combination of HCQ and PAR, either in their mixed form or when combined with other medications, have been reported in the literature. The quantification of HCQ and PAR was conducted using spectrophotometric techniques [11–17], Reversed-phase HPLC [18], TLC-densitometric methods [19, 20], UHPLC-MS Technique [21, 22], LC methods [23, 24], GC-mass [25] and potentiometric determination [26, 27].

Currently, there are no documented procedures available for the concurrent analysis of our mentioned drug combinations using recently developed techniques for resolving overlapping spectra. These techniques include zero order (dual wavelength, zero crossing, advanced absorption subtraction and spectrum subtraction), derivative (first derivative of zero crossing), ratio (ratio difference, ratio derivative) and mathematical (bivariate, simultaneous equation, and Q-absorbance) techniques, as far as our knowledge extends. As a result, the objective of this research is to produce additional straightforward, economical, precise, expeditious, and simple techniques for quantifying the aforementioned pharmaceutical compounds in combination, while minimizing the impact of excipients and additives in pharmaceutical formulations. Furthermore, the devised spectrophotometric methods are more precise and simpler to calculate than those previously reported.

## Experimental

### Apparatus

The Model 6800, a true double beam UV/visible spectrophotometer. Using the Jenway Model 6800 Flight Deck Software.

At room temperature, all measurements were performed in a 1 cm quartz cell within a wavelength range of 200–400 nm.

### Materials and reagents

#### Pure standards

Hydroxychloroquine and Paracetamol were acquired as a complimentary offering from Egyptian International Pharmaceutical Industries Co. (EIPICO), situated in 10th of Ramadan city, Egypt. Their purity was reported as 99.70% and 99.80%, respectively.

### Pharmaceutical formulations

Plaquenil® Tablets manufactured by SANOFI, Egypt, with the label claim of containing 200 mg of hydroxychloroquine were obtained from the market.

Panadol ®Tablets with a label claim of Paracetamol 500 mg were acquired from the market. They were manufactured by Glaxo Smithkline GSK, Egypt.

### Solvents

#### Distilled water

##### Standard solutions

HCQ and PAR stock standard solutions of 1000 µg /mL were prepared in pure distilled water. While 50 µg/mL HCQ and PAR working standard solutions were generated through the process of dilution from the stock solution using purified distilled water.

##### Laboratory prepared mixtures

By precisely transferring measured amounts from their standard solutions into 10 mL volumetric flasks and subsequently diluting with distilled water, solutions with various ratios of HCQ & PAR were created.

### Procedures

#### Construction of calibration curves

To generate working solutions of HCQ, aliquots of HCQ working standard solution (50 µg/mL) were added to a series of 10 mL volumetric flasks. The aliquots used were 0.6, 0.8, 1, 1.2, 1.4, 1.5, 1.8, 2, 2.5, 3, 3.5, 4, 4.5, 4.8, and 5 mL. The flasks were then diluted with distilled water to get working solutions with concentrations ranging from 3 to 25 µg/mL.

For PAR: 2–35 µg/mL working solutions were prepared by the addition of aliquots (0.4, 0.5, 1, 1.2, 1.5, 1.8, 2, 2.5, 2.8, 3, 3.5, 3.8, 4, 4.2, 4.5, 4.8, 5, 5.5, 6, 6.5, 7 mL) of PAR working standard solution (50 µg/mL) to 10 mL separate volumetric flasks series and diluting with distilled water.

The individual solutions were subjected to independent scanning, and the absorption spectra were measured at room temperature within the wavelength range of 200–400 nm for all measurements.

#### *For Zero crossing* spectrophotometric method

**Fig. 3:**
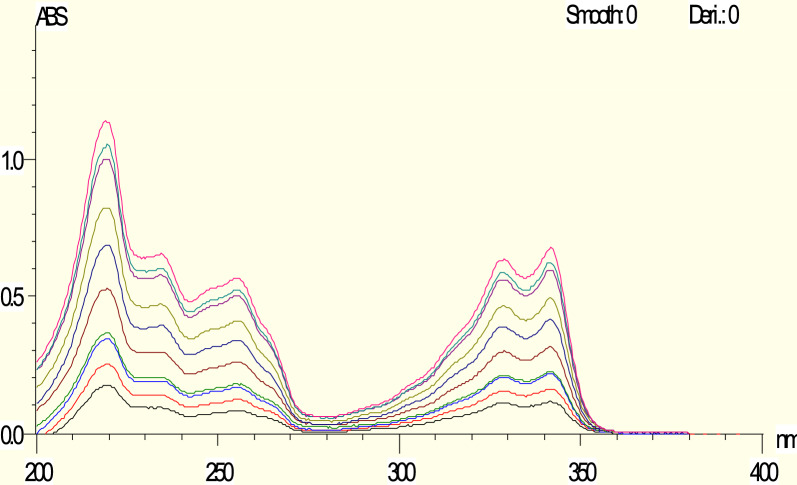
Zero absorption spectra of HCQ measured at 329 nm

The amplitude values were taken at a wavelength of 329 nm to determine the concentration of HCQ in the presence of PAR, where the absorbance of PAR is zero. (Fig. 3).

### *For Spectrum Subtraction method*

**Fig. 4:**
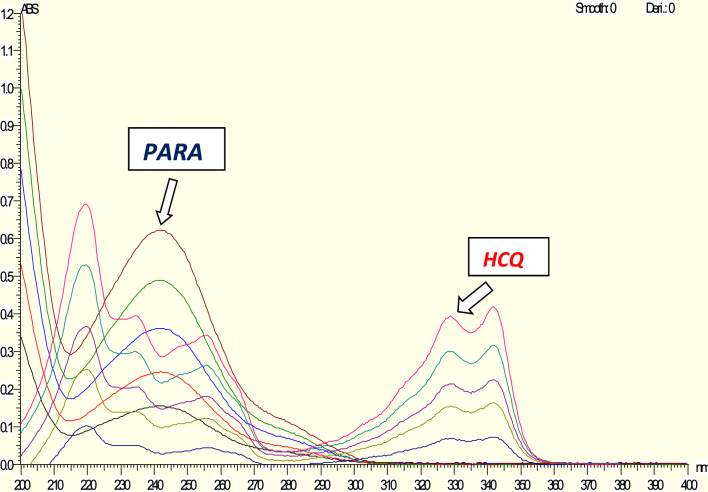
Zero absorption spectra of HCQ overlaid with zero absorption spectra of PAR showing that 220 and 242.5 are the maximum wavelengths for HCQ and PAR, respectively, and showing that 215.5 and 261.5 have the same absorbance in PARA, so we can measure HCQ absorption and 215.5 and 227.5 have the same absorbance in HCQ, so we can measure PAR absorption

Spectrum Subtraction method [28, 29] was used in HCQ determination in presence of PAR. Absorbance was calculated at wavelength 220 nm (Fig. 4) which is the maximum wavelength of HCQ after subtraction of PAR spectra from laboratory mixture of two drugs. Conversely, PAR was measured in the presence of HCQ. Absorbance was calculated at 242.5 nm (Fig. 4) which is the maximum wavelength of PAR after subtraction of HCQ spectra from laboratory mixture of two drugs.

### For dual wavelength method

The dual wavelength technique was employed to quantify the concentration of HCQ in the presence of PAR, as well as to determine the concentration of PAR in the presence of HCQ. The absorbance was measured at wavelengths of 215.5 nm and 261.5 nm, where the absorbance difference of PAR is zero. The concentration of HCQ may be estimated using the regression equation. Alternatively, the absorbance was recorded at wavelengths of 227.5 and 215 nm, where the difference in absorbance of HCQ is negligible (Fig. 4). The value of PAR can be calculated by applying the regression equation [30, 31].

#### Simultaneous equation method

Simultaneous equation method [32] uses the absorbance of two selected wavelengths, from the overlain spectra (Fig. 4) 220 nm (Kmax of HCQ) and 242.5 nm (Kmax of PAR) were selected for the formation of simultaneous equation. The A (1%, 1 cm) was determined at both the wavelengths selected for each drug. A set of two simultaneous equations was formed as:

$$\mathrm{Cx }= ({\text{A}}2{\text{ay}}1-\mathrm{ A}1{\text{ay}}2) ({\text{A}}2{\text{ay}}1-\mathrm{ A}1{\text{ay}}2)/ ({\text{ax}}2{\text{ay}}1-\mathrm{ ax}1{\text{ay}}2) ({\text{ax}}2{\text{ay}}1-\mathrm{ ax}1{\text{ay}}2)$$

$${\text{Cy}}= ({\text{A}}1{\text{ax}}2 -{\text{A}}2{\text{ax}}1) ({\text{A}}1{\text{ax}}2-\mathrm{ A}2{\text{ax}}1)/ ({\text{ax}}2{\text{ay}}1-\mathrm{ ax}1{\text{ay}}2) ({\text{ax}}2{\text{ay}}1 -{\text{ax}}1{\text{ay}}2)$$

where, A1 and A2 are the absorbance of sample solutions at 220 and 242.5 nm, respectively.

ax1 and ax2 (0.0881, 0.0339) are E (1%, 1 cm) of HCQ at 220 and 242.5 nm. ay1 and ay2 (0.0419, 0.0521) are E (1%, 1 cm) of PAR at 220 and 242.5 nm. Cx and Cy are concentrations of HCQ and PAR in mg/mL in sample solution. The values of Cx and Cy were calculated by putting the values of A1 and A2 to solve the simultaneous Eqs.

### For bivariate method

The formula (A Ai = m Ai. CA + e Ai) represents the linear calibration regression function employed in spectrophotometry to determine the analyte A at a specific wavelength (i). In this formula, C represents the concentration, m denotes the slope of the linear regression, and e represents the intercept value. If measurements are taken for the binary mixture (A, B) at two selected wavelengths (λ1, λ2), we will obtain two sets of equations:

$${{\text{A}}}_{{\text{AB}}1}= {{\text{m}}}_{{\text{A}}1}.{{\text{C}}}_{{\text{A}}} + {{\text{m}}}_{{\text{B}}1} . {{\text{C}}}_{{\text{B}}} + {{\text{e}}}_{{\text{AB}}1}$$

$${{\text{A}}}_{{\text{AB}}2}= {{\text{m}}}_{{\text{A}}2}. {{\text{C}}}_{{\text{A}}} + {{\text{m}}}_{{\text{B}}2} . {{\text{C}}}_{{\text{B}}} + {{\text{e}}}_{{\text{AB}}2}$$

where e _AB1_ and e _AB2_ are the sum of the intercepts at the chosen two wavelengths (e _ABi_ = e _Ai_ + e _Bi_) then the values of C_A_ & C_B_ can be calculated as follows:

$${{\text{C}}}_{{\text{B}}} = {{\text{m}}}_{{\text{A}}2}({{\text{A}}}_{{\text{AB}}1}- {{\text{e}}}_{{\text{AB}}1}) + {{\text{m}}}_{{\text{A}}1} ({{\text{e}}}_{{\text{AB}}2}-{{\text{A}}}_{\mathrm{ AB}2}) / {{\text{m}}}_{{\text{AB}}1} - {{\text{m}}}_{{\text{A}}1} {{\text{m}}}_{{\text{B}}2}$$

$${{\text{C}}}_{{\text{A}}}= {{\text{A}}}_{{\text{AB}}1}- {{\text{e}}}_{{\text{AB}}1}- {{\text{m}}}_{{\text{B}}1}\cdot {{\text{C}}}_{{\text{B}}}/ {{\text{m}}}_{{\text{A}}1}$$

By selecting two specific wavelengths and utilizing the parameters of linear regression to identify each compound at those wavelengths, these simple procedures can facilitate the differentiation of the binary mixture. The best wavelengths can be found using the Kaiser method (Additional file 1: Table S1). For every combination of binary mixture and pair of selected wavelengths, a sequence of sensitivity matrices K is calculated: where m_A1,2_ are the slopes (sensitivity parameters) of component A and m_B1,2_ are the slopes (sensitivity parameters) of component B. The resolution and determinants of these matrices were calculated. The collection of wavelengths with the maximum absolute matrix determinant was selected [33]. At the same wavelengths (226 and 236 nm), the bivariate approach was used to determine HCQ and PAR in the presence of each other (Fig. 4).

For Q Absorbance method.

**Fig. 5:**
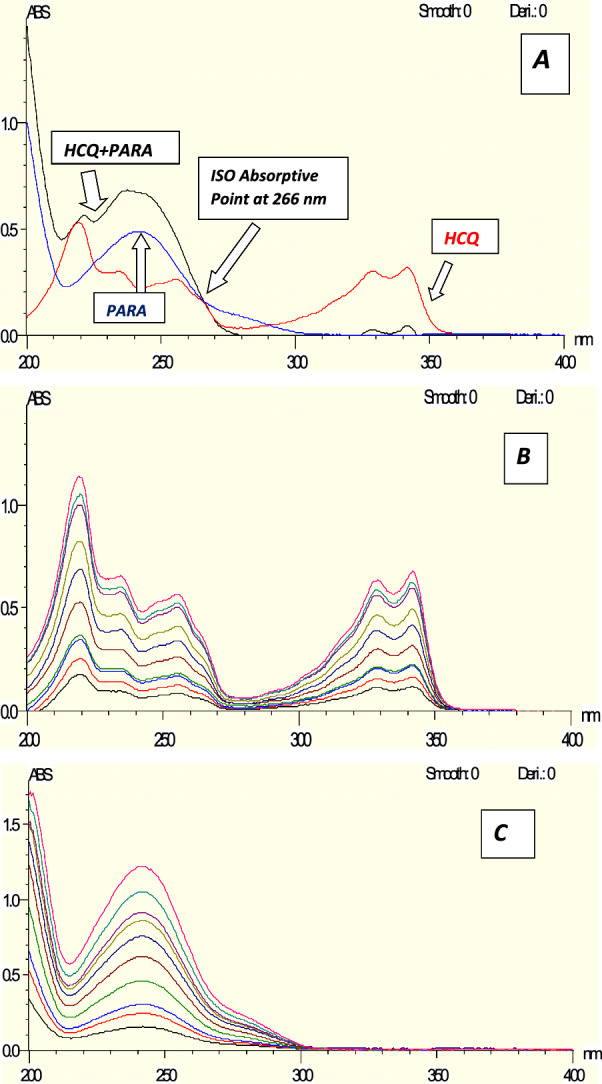
**A** Zero absorption spectrum of 8 µg/ml HCQ overlaid with 8 µg/mL PARA and a mixture of 4µg/mL HCQ & 4 µg/mL PARA revealed that 266 nm is an Iso absorptive point and revealed that 266 and 313.5 nm has the same absorbance in HCQ. **B** Zero absorption spectra of HCQ measured at 220 nm. **C** Zero absorption of PARA measured at 242.5 nm

Q Absorbance method uses the ratio of absorbance at two selected wavelengths, one, which is an iso absorptive point and other being the λmax of one of the two components. From the overlay spectra of the two drugs, it is evident that HCQ and PAR show an iso absorptive point at 266 (Fig. 5A) nm and the second wavelength used is 220 nm, which is the λmax of HCQ (Fig. 5B). The absorbances at 266 nm (iso absorptive point) and 220 nm (λmax of HCQ) were measured and absorptivities were calculated. The concentration of two drugs in the mixture can be calculated using following equations:

$$\mathrm{Cx }= \left({\text{Qm}}-{\text{Qy}}\right).\mathrm{ A}1/\left({\text{Qx}}-{\text{Qy}}\right).\mathrm{ ax}1$$

$$\mathrm{Cy }= ({\text{Qm}}-{\text{Qx}}).\mathrm{ A}1 / ({\text{Qy}}-{\text{Qx}}).\mathrm{ ay}1$$

where

$$\mathrm{Qm }={\text{A}}2/{\text{A}}1$$

$$\mathrm{Qx }=\mathrm{ ax}2/\mathrm{ ax}1$$

$$\mathrm{Qy }=\mathrm{ ay}2/\mathrm{ ay}1$$

A2 = Absorbance of Mixture at 220 nm; A1 = Absorbance of Mixture at 266 nm; ax1 = absorptivity of HCQ at 266 nm; ay1 = absorptivity of PAR at 266 nm; ax2 = absorptivity of HCQ at 220 nm; ay2 = absorptivity of PAR at 220 nm.

### For advanced absorption subtraction method

PAR was calculated using advanced absorption subtraction [30, 34] in the presence of HCQ. The absorbance was determined at wavelengths of 266 and 313.5 nm. The 266 nm wavelength was selected as an iso absorptive point, which allowed for the calculation of the total concentration (Fig. 5A). The absorbance of HCQ is the same for wavelengths 266 nm and 313.5 nm, resulting in a difference in absorbance of zero (Fig. 5B), PAR may be calculated by using the regression equation (Fig. 5C).

*Zero crossing* First derivative spectrophotometric method (D1).

**Fig. 6:**
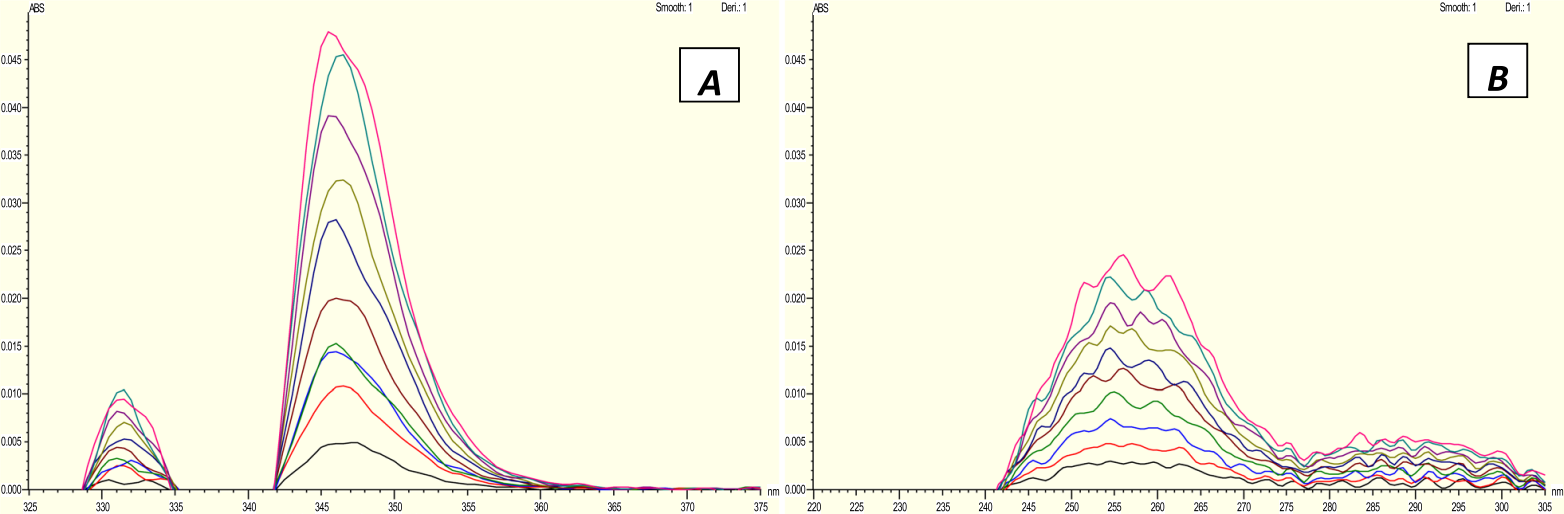
Zero Crossing Derivative spectra of **A** HCQ at 346 nm, **B** PARA at 255.5 nm

The first derivative corresponding to each absorption spectra was recorded. The amplitude values were measured at 346 and 255.5 nm for the purpose of determination of HCQ and PAR respectively to determine both HCQ (Fig. 6A) and PAR (Fig. 6B) in each other's presence [35].

### For ratio difference method

**Fig. 7:**
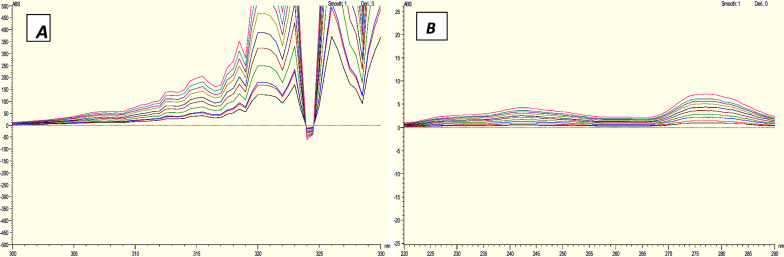
Ratio difference spectra of **A** HCQ using 10 µg/mL PARA as a divisor, **B** PARA using 10µg/mL HCQ as a divisor

The determination of HCQ and PAR was conducted using a ratio difference approach [36–38]. HCQ and PAR: The absorption spectra of HCQ at zero concentration were measured and stored. These spectra were then divided by the absorption spectrum of PAR at a concentration of 10 mg/mL (divisor). At 310.5 and 315 nm, the resultant spectra's amplitudes were measured, and the difference between them was calculated and used to create the calibration curve (Fig. 7A). On the other hand, the zero absorption spectra of PAR were captured and stored prior to being divided by the spectrum of 10 g/mL HCQ (divisor). The magnitudes of the resulting spectra were measured at 242.5 and 277.5 nm, and the disparity between them was computed and employed to construct the calibration curve. (Fig. 7B), after which the regression equation calculations were performed.

### For ratio derivative method

**Fig. 8:**
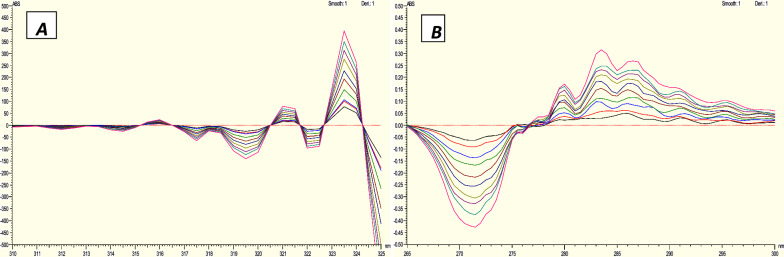
Ratio Derivative spectra of **A** HCQ using 10 µg/mL PARA as a divisor, **B** PARA using 10µg/mL HCQ as a divisor

The ratio derivative method was employed to determine the concentration of HCQ and PAR. The first derivative of the HCQ and PAR values that were previously recorded was computed using the ratio difference method. The amplitudes of the resultant spectra were then measured at a wavelength of 321.5 nm (Fig. 8A) for determination of HCQ and at 286 nm (Fig. 8B) In order to ascertain PAR, calibration curves were subsequently constructed for both medications. Following this, the calculations for the regression equation were performed.

### Evaluation of laboratory prepared mixtures

After preparing different ratios of mixtures in the laboratory, the spectra of these mixtures were evaluated and processed using the suggested methods.

#### Pharmaceutical formulation application

After weighing and crushing 5 Plaquenil® tablets, an amount equivalent to each tablet (200 mg HCQ) was combined to achieve a total concentration of 1000 mg HCQ. Similarly, 2 Panadol® tablets were weighed and crushed, and an amount equivalent to each tablet (500 mg PAR) was mixed to reach a total concentration of 1000 mg PAR. Subsequently, 50 mg of this mixture was used for further experimentation with conc (HCQ: PAR) is (1:1) into a 50 mL volumetric flask and diluted with distilled water as follows:

Initially, a volume of 30 mL of pure distilled water was introduced, sonicated, and then diluted to the proper concentration before filtering.

Second, to create a concentration equal to 100 µg/mL HCQ and PAR, 1 ml of the dilution was transferred into a 100 ml volumetric flask. Furthermore, any further dilutions were performed in 10 mL volumetric flasks and managed according to the prescribed procedures.

## Results and discussion

### Zero crossing spectrophotometric method

The absorbance was recorded at a wavelength of 329 nm, which allows for the determination of HCQ in the presence of PAR, as the absorbance of PAR at 329 nm is non-existent. The calibration curves demonstrated a valid linear correlation between concentrations and the wavelength throughout the range of 3–25 µg/mL for HCQ with a correlation coefficient of 0.9995. The approach demonstrated a high level of accuracy, with accepted values falling within the range of 99.56% ± 1.34. In addition, the technique specificity demonstrated accepted values falling within the range of 100.49% ± 1.44. The method is highly accessible, precise, and uncomplicated. However, its drawback lies in its limited applicability, as it can only be employed to determine HCQ (Table 1).

**Table 1:** Assay parameters and validation results obtained by applying the zero crossing, Spectrum Subtraction, Dual Wavelength and Advanced Absorption Subtraction spectrophotometric methods

Method Parameters	Zero Crossing	Spectrum Subtraction		Dual Wavelength		Advanced Absorption Subtraction
	HCQ	PARA	HCQ	HCQ	PARA	PARA
Wavelength (nm)	329	242.5	220	215.5–261.5	215–227.5	266–313.5
Linearity range (µg/mL) (n = 3)	3–25	2.5–25	1–25	3–25	2.5–35	2.5–35
Intercept	− 0.0127	0.0123	− 0.0482	− 0.0363	0.0096	0.0007
Slope	0.0405	0.0594	0.0740	0.0386	0.0162	0.0198
Correlation coefficient (r)	0.9995	0.9997	0.9997	0.9995	0.9991	0.9996
Accuracy (Mean ± SD)	99.56 ± 1.34	100.42 ± 0.74	99.39 ± 1.10	100.38 ± 0.59	101.64 ± 0.60	101.69 ± 0.11
*Precision (± %RSD)*						
Repeatability	100.35 ± 0.62	100.51 ± 0.40	100.04 ± 0.72	99.90 ± 1.12	101.33 ± 0.48	100.57 ± 0.57
Intermediate precision	99.11 ± 0.66	99.98 ± 0.73	99.67 ± 0.76	99.13 ± 1.37	100.35 ± 0.74	101.12 ± 0.81
Specificity (Mean ± SD)	100.49 ± 1.44	99.87 ± 0.90	99.43 ± 0.93	100.31 ± 1.26	99.32 ± 1.24	101.09 ± 0.65

#### For spectrum subtraction

Absorbance was measured at 220 nm, where the estimation of HCQ may be done in the presence of PAR. The calibration curves for HCQ demonstrated strong linear relationships between concentrations and spectrum subtraction in the range of 1–25 µg/mL, as indicated by a correlation coefficient of 0.9997. The method's accuracy was determined to be 99.39% ± 1.10, indicating that the authorized values fell within this range. In addition, the method specificity indicated that values of 99.43% ± 0.93 were considered acceptable. The results are demonstrated in Table 1. In contrast, the absorbance was determined at 242.5 nm, where PAR can be calculated when HCQ is present. With a correlation coefficient of 0.9997. The calibration curves demonstrated a clear linear relationship between concentrations and the dual wavelength within the 2.5–25 µg/mL range for PAR. The accuracy of the method was determined to be within the range of 100.42% ± 0.74, indicating that the authorized values were within this range. In addition, the approach specificity indicated that the accepted results ranged from 99.87% ± 0.90. The findings are illustrated in Table 1. Spectrum subtraction is a straightforward and uncomplicated method that does not necessitate any supplementary processing, as it depends just on zero absorption spectra. Acquiring the zero order spectra of the drug can be accomplished with a few simple procedures. However, the presence of noise interference hinders the accurate determination of the target drug concentration through subtraction.

#### The dual wavelength approach

The dual wavelength involved measuring the absorbance at specific wavelengths of 215.5 nm and 261.5 nm to identify the existence of HCQ in the presence of PAR. The calibration curves demonstrated a linear relationship between concentrations and the dual wavelength within the range of 3–25 µg/mL for HCQ, with a high correlation coefficient of 0.9995. The approach demonstrated a high level of accuracy, with data falling within the range of 100.38% ± 0.59. In addition, the method specificity demonstrated accepted values ranging from 100.31% to 101.57%. Alternatively, the absorbance was recorded at wavelengths of 227.5 nm and 215 nm, which allows for the determination of PAR in the presence of HCQ. The calibration curves demonstrated a valid linear connection between concentrations and the dual wavelength within the range of 2.5–35 µg/mL for PAR, with a high correlation coefficient of 0.9991. The approach demonstrated a high level of accuracy, with the measured values falling within the range of 101.64% ± 0.60. In addition, the technique specificity demonstrated accepted values falling within the range of 99.32% ± 1.24. The findings are disclosed in Table 1. The dual wavelength approach is a straightforward and precise technique that does not necessitate the use of specialized software applications or further processing. It is the fastest method to determine both drugs.

#### For advanced absorption subtraction approach

The advanced absorption subtraction method was employed to evaluate the absorbance of PAR in the presence of HCQ. Measurements were taken at 266 nm and 313.5 nm, with 266 nm selected as the iso-absorptive point. This allows for the simultaneous assessment of both PAR and HCQ in the presence of each other. PAR concentrations had a correlation value of 0.9996 and exhibited a linear relationship in the 2.5–35 µg/mL range. The technique displayed acceptable specificity values of 101.09% ± 0.65 and acceptable accuracy values of 101.69% ± 0.11 (Table 1). Although this approach is a straightforward and precise technique that does not necessitate the use of specialized software applications or further processing, it still slower than dual wavelength and need a specific condition to be applied.

#### For simultaneous equation method

Two wavelengths, 220 nm and 242.5 nm, corresponding to the maximum absorption of HCQ and PAR respectively, were employed to quantify the presence of HCQ and PAR in the same sample. The calibration curves demonstrated valid linear correlations between concentrations and absorbance throughout the respective ranges of 3–25 µg/mL for HCQ and 2.5–35 µg/mL for PAR with correlation coefficients of (0.9996,0.9996) for HCQ and of (0.9996,0.9991) for PAR for the two wave lengths. The approach demonstrated a high level of accuracy by aligning with accepted values (99.85% ± 0.72, 100.62% ± 1.22) for HCQ and (100.47% ± 1.35, 100.39% ± 1.12) for PAR for the two wave lengths. The specificity of the methods demonstrated accepted values with 100.24% ± 1.29 for HCQ and 99.05% ± 0.44 for PAR. The method is very basic, accurate, and simple. The sole restriction of this method is that it requires some special calculations to solve the simultaneous Eqs. The results are detailed in Table 2.

**Table 2:** Assay parameters and validation results obtained by applying the Simultaneous Equation, Bivariate and Q – Absorbance spectrophotometric methods

Method parameters	Simultaneous equation				Bivariate				Q-Absorbance			
	HCQ		PARA		HCQ		PARA		HCQ		PARA	
Wavelength (nm)	220	242.5	220	242.5	226	236	226	236	266	220	226	220
Linearity range (µg/mL) (n = 3)	3–25	3–25	2.5–35	2.5–35	3–25	3–25	2.5–35	4–35	3–25	3–25	2.5–35	2.5–35
Intercept	− 0.0513	− 0.0406	0.0009	0.0496	− 0.0419	− 0.0425	0.0118	0.0461	− 0.0160	− 0.0513	0.0004	0.0009
Slope	0.0742	0.0329	0.0336	0.0554	0.0452	0.0427	0.0427	0.0534	0.0208	0.0742	0.0202	0.0336
Correlation coefficient (r)	0.9996	0.9996	0.9996	0.9991	0.9996	0.9996	0.9997	0.9992	0.9997	0.9996	0.9996	0.9996
Accuracy (Mean ± SD)	99.58 ± 0.72	100.62 ± 1.22	100.47 ± 1.35	100.39 ± 1.12	99.94 ± 1.25	100.14 ± 1.11	102.80 ± 1.07	102.27 ± 0.69	101.15 ± 1.41	99.35 ± 1.10	100.18 ± 0.98	101.46 ± 0.48
*Precision (± %RSD)*												
Repeatability	100.57 ± 0.89		99.97 ± 0.73		100.45 ± 1.15		101.35 ± 0.63		100.88 ± 0.63		100.40 ± 0.66	
Intermediate precision	98.97 ± 0.65		100.79 ± 0.62		90.26 ± 7.01		101.92 ± 1.36		99.01 ± 0.86		100.70 ± 0.64	
Specificity (Mean ± SD)	100.24 ± 1.29		99.05 ± 0.44		99.75 ± 1.46		101.01 ± 1.53		101.26 ± 0.70		100.27 ± 1.01	

#### For bivariate method

In the context of statistical analysis, the term "bivariate method" refers to a technique that involves the examination and analysis of two variables simultaneously. The absorbance was measured at 226 and 236 nm, allowing for the simultaneous evaluation of HCQ and PAR utilizing these wavelengths. The calibration curves exhibited satisfactory linear correlations between concentrations and the bivariate in the range of 3–25 µg/mL for HCQ and 2.5–35 µg/mL range for PAR, with correlation coefficients of 0.9996 and 0.9996 for HCQ and 0.9997 & 0.9992 for PAR. The method's accuracy showed approved values of 99.94% ± 1.25 & 100.14 ± 1.11 for HCQ and 102.80% ± 1.07 and 102.27 ± 0.69 for PAR. The method specificity revealed accepted values with 99.75% ± 1.46 for HCQ and 100.01% ± 1.53 for PAR. The results are illustrated in Table 2.

The bivariate procedures, similar to the ones mentioned above, are fundamental, precise, and straightforward. The only limitation of this method is that it necessitates preliminary computations, specifically the implementation of the Kaiser method, in order to determine the ideal two wavelengths which make it more accurate than simultaneous equation. The results of Kaiser Method are recorded in Additional file 1: Table S1.

### Q absorbance method

The absorbance at wavelengths of 266 nm and 220 nm were utilized to determine the concentrations of HCQ and PAR in the presence of each other. The calibration curves demonstrated valid linear correlations between concentrations and absorbance throughout the range of 3–25 µg/mL for HCQ and 2.5–35 µg/mL for PAR with correlation coefficients of 0.9997 & 0.9996 for HCQ and of 0.9996 for PAR. The accuracy of the method illustrated accepted values with 101.15% ± 1.41 & 99.35% ± 1.10 for HCQ and 100.18% ± 0.98 & 101.46 ± 0.48 for PAR. The specificity of the methods demonstrated accepted values with 101.26% ± 0.70 for HCQ and 100.27% ± 1.01 for PAR. The results are detailed in Table 2. Q Absorbance is very easy and simple as it depends on zero absorption spectra without the need of extra processing. However, there are two constraints to consider. Firstly, particular calculations are required to calculate the values of Q Absorbance. Secondly, completing the standard addition on each mixture takes more time.

#### Zero crossing first derivative spectrophotometric method (D1)

The developed derivative method depends on taking first derivative for both drugs HCQ and PAR and measuring the drug of interest at peak while another drug is crossing zero as shown in Fig. 5. In this work the amplitude values were measured at 346 and 255.5 nm for determination of HCQ and PAR first order derivative, respectively. The calibration curves exhibited satisfactory linear correlations within the concentration range of 3–22 µg/mL for HCQ and 2.5–35 µg/mL for PAR. The correlation coefficient was found to be 0.9990 for HCQ and 0.9993 for PAR.The method's accuracy showed approved values of 99.84% ± 1.17 for HCQ and 99.32% ± 0.92 for PAR. The method specificity revealed accepted values with 100.33% ± 0.95 for HCQ and 100.94% ± 0.81 for PAR. Zero crossing derivative method is very easy, accurate, and simple and can overcome the limitation of zero crossing method which determine HCQ only. The results are documented in Table 3.

**Table 3:** Assay parameters and validation results obtained by applying the zero crossing derivative, ratio difference and ratio derivative spectrophotometric methods

Method parameters	Zero crossing derivative		Ratio difference		Ratio derivative	
	PARA	HCQ	PARA	HCQ	PARA	HCQ
Wavelength (nm)	255.5	346	277.5–242.5	315–310.5	286	321.5
Linearity range (µg/mL) (n = 3)	2.5–35	3–22	4–20	3–25	4–25	3–25
Intercept	0.0006	− 0.0004	0.0272	− 3.0589	0.0068	− 5.5719
Slope	0.0011	0.0028	0.1397	6.7181	0.0156	9.7111
Correlation coefficient (r)	0.9993	0.9990	0.9994	0.9997	0.9990	0.9995
Accuracy (Mean ± SD)	99.32 ± 0.92	99.84 ± 1.16	100.65 ± 0.68	99.63 ± 1.26	99.27 ± 1.23	99.48 ± 0.82
*Precision (± %RSD)*						
Repeatability	99.77 ± 0.79	100.39 ± 1.29	100.19 ± 0.87	100.66 ± 0.62	100.39 ± 1.11	100.05 ± 1.12
Intermediate precision	99.86 ± 0.77	99.04 ± 1.00	100.13 ± 0.82	99.3 ± 1.34	100.80 ± 0.64	99.93 ± 1.45
Specificity (Mean ± SD)	100.33 ± 0.95	100.94 ± 0.81	100.88 ± 0.62	100.54 ± 0.74	99.50 ± 1.20	100.82 ± 0.90

#### For ratio difference method

The Ratio difference approach involved the calculation of HCQ by measuring the absorbance at 310.5 and 315 nm, using10µg/mL PAR as a divisor. Alternatively, by employing a concentration of 10 µg/mL of HCQ as a divisor, the absorbance was assessed at wavelengths of 242.5 and 277.5 nm in order to ascertain the PAR. With a correlation coefficient of 0.9997 for HCQ and a correlation coefficient of 0.9994 for PAR. The calibration curves exhibited significant linear relationships between concentrations and the ratio difference within the range of 3–25 µg/mL for HCQ and 2.5–20 µg/mL for PAR. The method accuracy revealed accepted values of 99.63% ± 1.26 for HCQ and 100.65% ± 0.68 for PAR. Also, the method specificity revealed accepted values of 100.54% ± 0.74 for HCQ and 100.88% ± 0.62 for PAR. The results are demonstrated in Table 5. As previously said, the ratio difference approach is a Straightforward and precise method because it doesn't call for any special software. The sole limitation of this procedure is that it requires dividing the spectrum of the target compound by a specific divisor of the other compound and performing multiple tests to identify the most effective divisor. The results are documented in Table 3.

#### The ratio derivative approach

The absorbance was measured at a wavelength of 321.5 nm for the determination of HCQ and at a wavelength of 286 nm for the determination of PAR. The calibration curves demonstrated valid linear correlations between concentrations and the ratio derivative within the range of 3–25 µg/mL for HCQ and 4–25 µg/mL for PAR. The approach demonstrated a high level of accuracy, with HCQ yielding accepted values of 99.48% ± 0.82 and PAR yielding acceptable values of 99.72% ± 1.23. Similarly, the specificity of the methods was also within the accepted range, with HCQ showing values of 100.82% ± 0.90 and PAR showing values of 99.50% ± 1.20. While the ratio derivative approach is both straightforward and accurate, it requires an additional step to obtain the derivative of the ratio difference spectra. The results are documented in Table 3.

### Method validation

As per the ICH requirements, validation was conducted for each technique [39]. The linear regression results of the calibration curves indicated a strong linear correlation (Tables 1, 2 and 3).

As demonstrated in Tables 1, 2 and 3, the accuracy was assessed by conducting three replicates of a single concentration, resulting in good outcomes.

The specificity of the approaches was determined by evaluating the laboratory-prepared mixes of HCQ and PAR within the linearity range, resulting in positive findings (Tables 1, 2 and 3).

An investigation on the effects of three distinct drug concentrations the intra- and inter-day precisions can be calculated by doing three measurements on the same day and for three successive days (Tables 1, 2 and 3).

### Pharmaceutical formulation application

To determine HCQ & PAR in pharmaceutical formulations (Plaquenil® tablets) and (Panadol® tablets), the suggested procedures were successfully applied. The outcomes were respectable and sufficiently in line with the amounts indicated on the labels. The standard addition method was used, and the results revealed that there was no excipient interference (Tables 4, 5 and 6).

**Table 4:** Analysis of the pharmaceutical preparation (Plaquenil® tablets) by applying the Zero Crossing method, dual wavelength, Ratio difference method, and ratio derivative spectrophotometric methods

	Zero crossing method				Dual wavelength method								Ratio difference method			
	HCQ				HCQ				PAR				HCQ			
			Recovery%				Recovery%				Recovery%				Recovery%	
	Tablet Taken (µg/mL)	Standard Added (µg/mL)	Tablet	Added	Tablet Taken (µg/mL)	Standard Added (µg/mL)	Tablet	Added	Tablet Taken (µg/mL)	Standard Added (µg/mL)	Tablet	Added	Tablet Taken (µg/mL)	Standard Added (µg/mL)	Tablet	Added
	10	5	101.16	100.97	10	5	100.88	101.47	10	5	99.21	100.40	10	5	100.88	99.93
		10	100.39	101.67		10	101.21	99.62		10	101.68	101.44		10	101.75	99.68
		15	99.65	99.92		15	101.65	101.00		15	101.37	99.83		15	98.07	101.16
Mean			100.40	100.85			101.25	100.70			100.75	100.56			100.32	100.26
SD			0.75	0.88			0.39	0.96			1.34	0.82			1.93	0.79

	Ratio difference method				Ratio derivative method							
	PAR				HCQ				PAR			
			Recovery%				Recovery%				Recovery%	
	Tablet Taken (µg/mL)	Standard Added (µg/mL)	Tablet	Added	Tablet Taken (µg/mL)	Standard Added (µg/mL)	Tablet	Added	Tablet Taken (µg/mL)	Standard Added (µg/mL)	Tablet	Added
	10	5	101.34	98.36	10	5	99.01	100.55	10	5	101.57	98.45
		10	100.18	98.56		10	98.24	101.02		10	100.23	99.15
		15	99.60	99.10		15	98.08	101.48		15	99.66	100.71
Mean			100.37	98.68			98.44	101.02			100.49	99.44
SD			0.89	0.38			0.50	0.46			0.98	1.16

**Table 5:** Analysis of the pharmaceutical preparation (Plaquenil® tablets) by applying the advanced absorption subtraction method, spectrum subtraction method, Zero Crossing derivative and Bivariate spectrophotometric methods

	Advanced absorption subtraction method				Spectrum subtraction method								Zero crossing derivative method			
	PAR				HCQ				PAR				HCQ			
			Recovery%				Recovery%				Recovery%				Recovery%	
	Tablet Taken (µg/mL)	Standard Added (µg/mL)	Tablet	Added	Tablet Taken (µg/mL)	Standard Added (µg/mL)	Tablet	Added	Tablet Taken (µg/mL)	Standard Added (µg/mL)	Tablet	Added	Tablet Taken (µg/mL)	Standard Added (µg/mL)	Tablet	Added
	10	5	98.92	99.21	10	5	99.64	99.26	10	5	99.31	99.03	10	5	102.01	100.52
		10	101.81	98.76		10	101.19	98.03		10	100.66	100.64		10	101.65	99.80
		15	101.15	98.42		15	98.96	99.23		15	99.66	100.86		15	101.30	100.10
Mean			100.63	98.80			99.93	98.84			99.88	100.17			101.65	100.14
SD			1.51	0.40			1.14	0.70			0.70	1.00			0.36	0.36

	Zero Crossing derivative method				Bivariate method							
	PAR				HCQ				PAR			
			Recovery%		Recovery%						Recovery%	
	Tablet Taken (µg/mL)	Standard Added (µg/mL)	Tablet	Added	Tablet Taken (µg/mL)	Standard Added (µg/mL)	Tablet	Added	Tablet Taken (µg/mL)	Standard Added (µg/mL)	Tablet	Added
	10	5	100.24	99.45	10	5	98.34	99.59	10	5	98.18	99.39
		10	99.32	101.17		10	98.15	99.74		10	98.43	100.38
		15	98.03	98.54		15	98.05	101.91		15	98.60	98.03
Mean			99.20	99.72			98.18	100.20			98.40	99.26
SD			1.11	1.33			0.15	1.03			0.21	1.18

**Table 6:** Analysis of the pharmaceutical preparation (Plaqunil® tablets) by applying the Simultaneous equation and Q-absorbance spectrophotometric methods

	Simultaneous equation method								Q-absorbance method							
	HCQ				PAR				HCQ				PAR			
			Recovery%				Recovery%				Recovery%					
	Tablet Taken (µg/mL)	Standard Added (µg/mL)	Tablet	Added	Tablet Taken (µg/mL)	Standard Added (µg/mL)	Tablet	Added	Tablet Taken (µg/mL)	Standard Added (µg/mL)	Tablet	Added	Tablet Taken (µg/mL)	Standard Added (µg/mL)	Tablet	Added
	10	5	101.87	98.88	10	5	98.71	101.34	10	5	101.98	101.01	10	5	101.19	100.86
		10	99.91	101.24		10	98.07	101.47		10	100.49	100.41		10	101.89	98.30
		15	98.98	99.62		15	100.01	100.92		15	99.69	101.84		15	99.77	99.07
Mean			100.26	100.51			98.93	101.24			100.72	100.48			100.95	99.41
SD			1.48	1.21			0.99	0.29			1.16	0.72			1.08	1.31

## Conclusion

Hydroxychloroquine and Paracetamol in their combination medicinal dose forms were determined using zero order (dual wavelength, zero crossing, advanced absorption subtraction and spectrum subtraction), derivative (first derivative of zero crossing), ratio (ratio difference, ratio derivative) and mathematical (bivariate, simultaneous equation, and Q-absorbance) methods. All of the recommended processes can be effectively implemented for routine analysis using low-tech equipment or technology due to their simplicity, directness, accuracy, and sensitivity. Through a comparison of previous approaches, it was demonstrated that only the ratio difference and ratio derivative methods requires additional processing, while the other methods do not. The best and the fastest method is the dual wavelength method.

Using Distilled Water as solvent considered as green analytical chemistry as it environmentally friendly and no need for organic solvent [40].

### Supplementary Information

**Additional file 1.** **Table S1:** Application of the Kaiser method for the selection of wavelength set for the mixture of HCQ and PARA.

## Data Availability

The authors confirm that the data supporting the findings of this study are available within the manuscript, figures and tables files.

## References

[1] Martinez-Rivera RN, Taype-Rondan A (2020). Overmedication in COVID-19 Context: A Report From Peru. J Clin Pharmacol.

[2] Tan SHS, Hong CC, Saha S, Murphy D, Hui JH (2020). Medications in COVID-19 patients: summarizing the current literature from an orthopaedic perspective. Int Orthop.

[3] Mannaa IM, El Gazayerly ON, Abdelbary AA, Saleh SS, Mostafa DA. Validated green spectroscopic manipulation of area under the curve (AUC) for estimation of Simvastatin: Application to nano-structured lipid carriers and niosomal systems. 2023.

[4] Sebaiy MM, Sobhy M, Mattar AA (2020). Different techniques for overlapped UV spectra resolution of some co-administered drugs with paracetamol in their combined pharmaceutical dosage forms. Spectrochim Acta Part A Mol Biomol Spectrosc.

[5] Seligmann H (2021). Balanced evaluation of preliminary data on a candidate COVID-19 hydroxychloroquine treatment. Int J Antimicrob Agents.

[6] Baldia PH, Wernly B, Flaatten H, Fjølner J, Artigas A, Pinto BB, Schefold JC, Kelm M, Beil M, Bruno RRJB (2022). The association of prior paracetamol intake with outcome of very old intensive care patients with COVID-19: results from an international prospective multicentre trial. Tere.

[7] Crighton AJ, McCann CT, Todd EJ (2020). Safe use of paracetamol and high-dose NSAID analgesia in dentistry during the COVID-19 pandemic. Br Dental.

[8] Sestili P, Fimognari C. Paracetamol use in COVID-19: friend or enemy? 2020.

[9] Noureddine O, Issaoui N, Medimagh M, Al-Dossary O, Marouani H (2021). Quantum chemical studies on molecular structure, AIM, ELF, RDG and antiviral activities of hybrid hydroxychloroquine in the treatment of COVID-19: Molecular docking and DFT calculations. J King Saud Univ Sci.

[10] Geleris J, Sun Y, Platt J, Zucker J, Baldwin M, Hripcsak G, Labella A, Manson DK, Kubin C, Barr RG (2020). Observational Study of Hydroxychloroquine in Hospitalized Patients with Covid-19. N Engl J Med.

[11] Singh A, Sharma PK, Gupta R, Mondal N, Kumar S, Kumar M. Development and validation of UV-spectrophotometric method for the estimation of hydroxychloroquine sulphate. 2016.

[12] Rajitha K, Prasanna NL, Vasundhara G, Kumar RN, Kumar AA (2014). UV spectrophotometric method development and validation for the simultaneous quantitative estimation of mebeverine hydrochloride and chlordiazepoxide in capsules. Int J Pharm Pharm Sci.

[13] Bhati KN, Mashru R (2022). Development and validation of extractive spectrophotometric method for estimation of hydroxychloroquine sulphate by using smartphone application. J Drug Deliv Ther.

[14] Desta K, Amare M (2017). Validated UV-visible spectrometry using water as a solvent for determination of chloroquine in tablet samples. Chem Int.

[15] Behera S, Ghanty S, Ahmad F, Santra S, Banerjee S (2012). UV-visible spectrophotometric method development and validation of assay of paracetamol tablet formulation. J Anal Bioanal Techniques.

[16] Khaskheli AR, Shah A, Bhanger MI, Niaz A, Mahesar S (2007). Simpler spectrophotometric assay of paracetamol in tablets and urine samples. Spectrochim Acta Part A Mol Biomol Spectrosc.

[17] Shrestha BR, Pradhananga RR (2009). Spectrophotometric method for the determination of paracetamol. J Nepal Chem Soc.

[18] Sharma A, Ankalgi AD, Devi A, Pandit V, Ashawat MS (2021). Analytical method development and validation for simultaneous estimation of methotrexate and hydroxychloroquine sulfate in bulk drug by using RP-HPLC. Asian J Pharma Anal.

[19] Udosen E (1993). Determination of the percentage purity, active ingredients and other parameters in paracetamol and chloroquine syrups. Cent Afr J Med.

[20] El-Koussi WM, Atia NN, Saleh GA, Hammam N (2019). Innovative HPTLC method for simultaneous determination of ternary mixture of certain DMARDs in real samples of rheumatoid arthritis patients: an application of quality by design approach. J Chromatogr B.

[21] Duarte NJ, Kupa LV, Ferreira-Filho JC, Fontoura N, Chalom MY, Romano P, Ebner PA, Silva CA, Carvalho VM, Bonfá E (2021). UHPLC-MS/MS method for determination of hydroxychloroquine and its main metabolites in oral fluid and whole blood for therapeutic drug monitoring. J Appl Lab Med.

[22] Gamal S, Mohamed GG, Salih SA, Ezzeldin MI, Mandour AA (2023). Rapid and validated UHPLC method for simultaneous determination of sofosbuvir, ledipasvir and paracetamol as commonly repurposed drugs for COVID-19 treatment: application in spiked human plasma. Fut J Pharm Sci.

[23] Dongala T, Katari NK, Palakurthi AK, Katakam LNR, Marisetti VM (2020). Stability indicating LC method development for hydroxychloroquine sulfate impurities as available for treatment of COVID-19 and evaluation of risk assessment prior to method validation by quality by design approach. Chromatographia.

[24] Sultana N, Arayne MS, Ali SN. An ultra-sensitive LC method for the simultaneous determination of paracetamol, carbamazepine, losartan and ciprofloxacin in bulk drug, pharmaceutical formulation and human serum by programming the detector. 2013. 10.1002/jssc.201300104 23897845

[25] Bodur S, Erarpat S, Günkara ÖT, Bakırdere S (2021). Accurate and sensitive determination of hydroxychloroquine sulfate used on COVID-19 patients in human urine, serum and saliva samples by GC-MS. Journal of Pharmaceutical Analysis.

[26] Maia LFO, Reis EL, Reis C, Goncalves Reis CD, de Carvalho Damasceno OI (2019). Potentiometric determination of paracetamol in pharmaceutical formulations by the analyte pulse perturbation technique using Belousov-Zhabotinskii oscillating chemical reaction. J Anal Chem.

[27] Khalil MM, El-aziz GMA, Ashry A (2018). Potentiometric sensors based on hydroxychloroquine-phosphotungstate ion-pair and β-cyclodextrin ionophore for improved determination of hydroxychloroquine sulfate. J Iran Chem Soc.

[28] Samir A, Lotfy HM, Salem H, Abdelkawy M (2014). Development and validation of simultaneous spectrophotometric and TLC-spectrodensitometric methods for determination of beclomethasone dipropionate and salbutamol in combined dosage form. Spectrochim Acta Part A Mol Biomol Spectrosc.

[29] Lotfy H, Saleh S, Rostom Y, Obaydo R, Ahmed D (2023). Advanced approaches in green univariate spectrophotometric methods. Sustain Approach Pharma Sci.

[30] Sebaiy MM, Sobhy M (2020). Different techniques for overlapped UV spectra resolution of some co-administered drugs with paracetamol in their combined pharmaceutical dosage forms. Spectrochimica Acta Part A.

[31] Rostom Y, Lotfy HM, Öztürk M, Tiris G, Erk N, Saleh SS (2021). Trade-off efficacy and data processing strategy in the power of spectral resolution of co-formulated antihypertensive pharmaceuticals. Spectrochim Acta Part A Mol Biomol Spectrosc.

[32] Shetty PR (2014). Applications of simultaneous equation method and derivative method for the determination of rabeprazole sodium and levosulpiride in pharmaceutical dosage form and dissolution samples. Arab J Basic Appl Sci.

[33] Lataifeh A, Wedian F (2014). Bivariate Calibration Method vs. HPLC and Derivative Spectroscopy for Simultaneous Determination of Binary Drugs in Pharmaceutical Formulations: A study on Prifinium Bromide/Paracetamol and Amoxicillin/Potassium Clavulanate. Analyt Chem Lett.

[34] Lotfy HM, Saleh SS, Hassan NY, Salem H (2014). A comparative study of novel spectrophotometric methods based on isosbestic points; application on a pharmaceutical ternary mixture. Spectrochim Acta Part A Mol Biomol Spectrosc.

[35] Elmasry MS, Hassan WS, El-Mammli MY, Badrawy M (2022). Earth friendly spectrophotometric methods based on different manipulation approaches for simultaneous determination of aspirin and omeprazole in binary mixture and pharmaceutical dosage form: Comparative statistical study. Spectrochim Acta A Mol Biomol Spectrosc.

[36] Elmasry MS, Hassan WS, El-Mammli MY, Badrawy M (2022). Earth friendly spectrophotometric methods based on different manipulation approaches for simultaneous determination of aspirin and omeprazole in binary mixture and pharmaceutical dosage form: Comparative statistical study. Spectrochim Acta Part A Mol Biomol Spectrosc.

[37] Lotfy HM (2012). Comparative study of novel spectrophotometric methods manipulating ratio spectra: an application on pharmaceutical ternary mixture of omeprazole, tinidazole and clarithromycin. Spectrochim Acta Part A Mol Biomol Spectrosc.

[38] Saleh SS, Lotfy HM, Tiris G, Erk N, El-Naem OA (2022). The power of High Impact Amplitude Manipulation (HIAM) technique for extracting the basic spectra of two Fixed-dose combinations (FDC)-Spectrophotometric purity analysis via spectral contrast angle. Spectrochim Acta Part A Mol Biomol Spectrosc.

[39] Ich I (2005). Topic Q2 (R1) validation of analytical Procedures: text and methodology. Int Conf Harmon.

[40] Płotka-Wasylka JJT (2018). A new tool for the evaluation of the analytical procedure. Green Analyt Proc Index.

