# Supplementary material for: Various techniques for resolving overlapping ultraviolet spectra of combination pharmaceutical dosage forms containing hydroxychloroquine and paracetamol
Source: BMC Chem. 2024 May 28;18(1):104. doi: 10.1186/s13065-024-01187-2 (PMC11134631; doi:10.1186/s13065-024-01187-2)
Supplement: Supplementary file 1 — Additional file 1. Table S1: Application of the Kaiser method for the selection of wavelength set for the mixture of HCQ and PARA. [file 13065_2024_1187_MOESM1_ESM.docx]

Table S1: Application of the Kaiser method for the selection of wavelength set for the mixture of HCQ and PARA.

| **λ_1_**  **λ_2_** | **226 nm** | **228 nm** | **230 nm** | **232 nm** | **234 nm** | **236 nm** |
| --- | --- | --- | --- | --- | --- | --- |
| **226 nm** | 0 |  |  |  |  |  |
| **228 nm** | 202 | 0 |  |  |  |  |
| **230 nm** | 334 | 127 | 0 |  |  |  |
| **232 nm** | 428 | 216 | 88 | 0 |  |  |
| **234 nm** | 486 | 268 | 138 | 48 | 0 |  |
| **236 nm** | 590 | 372 | 244 | 157 | 112 | 0 |
